# A Fruitful Source of Epilepsy

**Published:** 1861-07

**Authors:** 


*A Fruitful Source of Epilepsy.*—Dr. Demereaux states, as we learn from the Maryland and Virginia Journal, that in a number of cases examined into, he has traced the cause of epilepsy in the offspring to the intoxication of the father at the time of sexual intercourse. He thinks that the baneful influence of drunkenness on the part of the parent on the fœtus, should be widely promulgated. It is certainly a strong argument, if arguments were needed, in favor of temperance.

*Hydrocyanate of Iron in Epilepsy.*—The Cincinnati Lancet and Observer says: "Dr. G. S. Baily, a retired physician of Iowa, states in a letter to the editors of the Journal of Materia Medica, that his only son, after having been treated six years for epilepsy with every remedy that medical skill could suggest, without success, was finally cured with the hydrocyanate of iron, by Prof. D. L. McGugin, of Keokuk. The formula employed corresponds with the one used by Dr. Treat (Cin. Lancet & Obs., June, 1860, p. 383): hydrocyanate of iron, one drachm; powder of valerian, two drachms; extract of Indian hemp, one drachm, being originally added by McGugin. Make into one hundred and twenty pills. One of them is to be taken three times a day, gradually increased to four."

*Glycerine as a Solvent for Belladonna.*—M. Foucher recommends the solution of belladonna in glycerine, instead of water, for the purpose of applying it around the brow to produce dilatation of the pupil. The extract is thus prevented from drying into a hard crust, and a greater certainty of absorption of the narcotic is insured.

[This would also be a good form for applying belladonna to the breast for the purpose of arresting the secretion of milk—one or two drachms of belladonna rubbed up in an ounce of glyceryne and smeared over the breast; the addition of thirty or forty grs. of camphor would be beneficial —Eds. St. Louis Journal.]

